# Red and Near-Infrared Absorbing Dicyanomethylene Squaraine Cyanine Dyes: Photophysicochemical Properties and Anti-Tumor Photosensitizing Effects

**DOI:** 10.3390/ma13092083

**Published:** 2020-05-01

**Authors:** Tiago D. Martins, Eurico Lima, Renato E. Boto, Diana Ferreira, José R. Fernandes, Paulo Almeida, Luis F. V. Ferreira, Amélia M. Silva, Lucinda V. Reis

**Affiliations:** 1 Chemistry Centre of Vila Real (CQ-VR), University of Trás-os-Montes and Alto Douro, Quinta de Prados, 5001-801 Vila Real, Portugal; tiago_dourado@hotmail.com (T.D.M.); eurico_lima@icloud.com (E.L.); jraf@utad.pt (J.R.F.); 2 Centre for Research and Technology of Agro-Environmental and Biological Sciences (CITAB-UTAD), University of Trás-os-Montes and Alto Douro, Quinta de Prados, 5001-801 Vila Real, Portugal; 3 Health Sciences Research Centre (CICS-UBI), University of Beira Interior, Av. Infante D. Henrique, 6201-001 Covilhã, Portugal; rboto@ubi.pt (R.E.B.); pjsa@ubi.pt (P.A.); 4 Institute of Bioengineering and Biosciences (iBB), Higher Technical Institute, University of Lisbon, Av. Rovisco Pais, 1049-001 Lisbon, Portugal; diana.ferreira@det.uminho.pt (D.F.); luisfilipevf@ist.utl.pt (L.F.V.F.); 5 Department of Biology and Environment, University of Trás-os-Montes and Alto Douro, Quinta de Prados, 5001-801 Vila Real, Portugal

**Keywords:** dicyanomethylene squaraine dyes, singlet oxygen, photostability, antiproliferative effects, photodynamic therapy

## Abstract

Photodynamic therapy is a medical modality developed for the treatment of several diseases of oncological and non-oncological etiology that requires the presence of a photosensitizer, light and molecular oxygen, which combined will trigger physicochemical reactions responsible for reactive oxygen species production. Given the scarcity of photosensitizers that exhibit desirable characteristics for its potential application in this therapeutic strategy, the main aims of this work were the study of the photophysical and photochemical properties and the photobiological activity of several dicyanomethylene squaraine cyanine dyes. Thus, herein, the study of their aggregation character, photobleaching and singlet oxygen production ability, and the further application of the previously synthesized dyes in Caco-2 and HepG2 cancer cell lines, to evaluate their phototherapeutic effects, are described. Dicyanomethylene squaraine dyes exhibited moderate light-stability and, despite the low singlet oxygen quantum yields, were a core of dyes that exhibited relevant *in vitro* photodynamic activity, as there was an evident increase in the toxicity of some of the tested dyes exclusive to radiation treatments.

## 1. Introduction

Photodynamic therapy is a successful and clinically approved medical technique used in the treatment of oncological and non-oncological diseases [1,2], which is based on the interaction between a photosensitizer compound, light stimuli and molecular oxygen [3]. When conjugated, these three components allow the production of type I and/or type II reactions, in which type II reactions are responsible for the production of singlet oxygen and type I the generation of other reactive oxygen species [4]. These cytotoxic species are the cause of biochemical effects causing oxidative damages, which will culminate in target cells’ death [5]. Photodynamic therapy is distinguished by its minimal invasion and reduced damage to the surrounding healthy tissue compared to the most current therapies in cancer treatment, such as chemotherapy and radiotherapy [6].

The discovery of novel, more efficient photosensitizers, presenting fewer side effects, namely squaraine cyanine dyes, has been explored by several research groups [7,8,9], since the photosensitizers hitherto marketed, mainly porphyrins and their derivatives, have several disadvantages, for instance, poor light absorption and cutaneous photosensitivity [10]. For this purpose, there are some required characteristics to be considered as an ideal photosensitizer, namely: the ability to selectively accumulate in the target tissue, light absorption at high wavelengths, more precisely within the “phototherapeutic window” (600–850 nm); to be innocuous in the absence of radiation; to have a high singlet oxygen quantum yield; and to be easily eliminated from the body [11,12].

Squaraine cyanine dyes, usually called squaraine dyes, are a family of compounds discovered in 1965 by Treibs and Jacob [13], whose suitable photochemical and photophysical properties give them the potential to be ideal for use in many biotechnological and biomedical applications, such as sensitizers for solar energy conversion [14,15], probes for metal ions and biomolecules detection [16,17,18,19,20], photodynamic therapy photosensitizers [7,8,9,21], among many others. The aforementioned dyes are known by their high molar extinction coefficient and also by their intense and narrow absorption bands in the visible and near-infrared regions due to the charge transfer system within the molecule. Moreover, the diversity of possible changes in the molecular structure of this group of dyes allows the research groups to shape and control this core of compounds to exhibit different properties according to the desired application [22]. The photosensitizing potential of squaraine dyes has already been proved, both in *in vitro* [7,21] and *in vivo* studies [23,24], showing that with the appropriate substituents, the ideal properties can be obtained to act as photodynamic therapy photosensitizers.

Although the synthesis of dicyanomethylene-substituted dyes is already known [25], reports on the preparation of novel dyes from this core of compounds are still ongoing, and the results of the studies of their potential application in technological [26] and biomedical areas [16,24,27] are very encouraging. Regarding its possible use as photosensitizers, Wei et al. [24] reported an *in vitro* and *in vivo* study in which this dye’s scaffold presents an intense phototherapeutic activity. The squaraines bearing the dicyanomethylene functional group exhibit inherent characteristics with those of an ideal photodynamic therapy photosensitizer, since when introduced in the four-membered central ring, it is the promotor that is responsible for their phototherapeutic activity [24].

**Figure 1:**
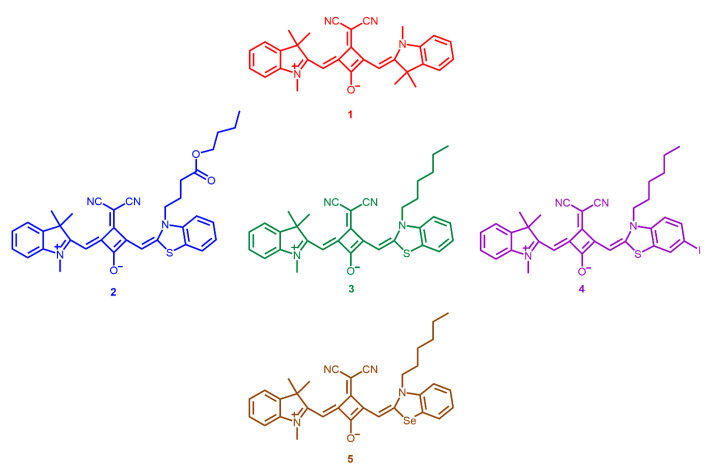
Dicyanomethylene squaraine dyes **1**–**5** involved in the present photodynamic study.

Regarding the dicyanomethylene squaraine cyanine dyes shown in Figure 1, their experimental details and spectroscopic characterization were recently published by us, as fluorescent probes for protein detection [16]. However, and as far as we know, the *in vitro* phototherapeutic effects presented by these dyes have never been tested, nevertheless, this study is assumed to be quite pertinent, given the encouraging results of some *in vitro* and *in vivo* works already reported [24] and the scantiness of photodynamic therapy studies related to this scaffold of compounds. The fact that these dyes have shown good interaction with serum albumin [16], according to some studies [28,29,30], also makes these compounds potential photodynamic therapy photosensitizers with increased phototherapeutic efficiency. Additionally, the choice to evaluate the *in vitro* photodynamic activity of indolenine-based squaraine dyes is due to the fact that this heterocycle, according to recent studies [7,31], provides desirable photophysical properties, such as excellent stability, as well as the high yield of photocytotoxicity. The evaluation of squaraine dyes with at least one benzoselenazole heterocyclic moiety is also of high relevance since it is reported as being capable of enhancing the ability to produce singlet oxygen of this scaffold of dyes, as a consequence of the so-called “heavy atom effect” [32]. Benzothiazole heterocyclic moiety is also of some relevance since some studies showed that they have great phototherapeutic ability, as well as good interaction with biomolecules [20,21].

## 2. Materials and Methods

### 2.1. Visible and Near-Infrared Absorption Spectra

The steady-state absorption spectra were recorded on a Perkin Elmer Lambda 25 spectrophotometer (Perkin Elmer, Waltham, MA, USA) using Perkin Elmer UV (ultraviolet) WinLab Data processor and Viewer software and in the spectral range of 500–750 nm at room temperature, and an ultraviolet quartz cuvette (1 cm path length; Hellma Analytics, Müllheim, Germany). These spectra were obtained using the following solvents: acetone, acetonitrile, dichloromethane, dimethylformamide, dimethyl sulfoxide, 1,4–dioxane, Dulbecco’s Modified Eagle Medium (DMEM) culture medium, ethanol, phosphate-buffered saline (PBS, pH 7.2), tetrahydrofuran and water. All organic solvents were obtained from commercial sources and used without further purification. Molar absorption coefficients were calculated by direct application of the Beer-Lambert law, using solutions of each squaraine dye (in the range of 10^−6^ to 10^−5^ M) performed in each solvent.

### 2.2. Singlet Oxygen Formation Quantum Yields Determination

The singlet oxygen measurement set-up was assembled in our laboratory. As an excitation source we used nitrogen pulsed laser OBB OL-401 (Horiba, NJ, USA) exciting at 337.1 nm. The detector is an indium gallium arsenide charged-coupled device (model iDus, Andor Technology Limited, Belfast, UK), working at (−60 °C), which was coupled to a fixed spectrograph. Further details regarding this technique can be found in reference [33]. By comparing the total area of the emission spectra for the reference compound (Phenazine in chloroform) and for each sample in the same solvent, the Φ_Δ_ values were obtained. An average of 100 phosphorescence spectra was used for each experimental determination.

### 2.3. Photostability Analysis

Dimethyl sulfoxide working solutions of each squaraine dye (**1**–**5**) and methylene blue (Merck, Darmstad, Germany) at a concentration of 15 µM were prepared. Dye solutions were irradiated with suitable light-emitting diode systems. The dyes absorbance was read at the maximum dimethyl sulfoxide absorption wavelength of each dye for 60 min using the microplate reader Multiscan Go spectrophotometer (Thermo Fisher Scientific, Vantaa, Finland).

### 2.4. In Vitro Phototherapeutic Potential Evaluation

#### 2.4.1. Cell Culture

The dyes’ photocytotoxicity was evaluated in two adherent cell lines: Caco-2 (human colorectal adenocarcinoma; Cell Lines Service, Eppelheim, Germany) and HepG2 (human hepatocellular carcinoma; American Type Culture Collection, Rockville, MD, USA). Cells were maintained in DMEM supplemented with 1 mM *L*-glutamine, 10% fetal bovine serum (FBS), 100 U/mL penicillin and 100 µg/mL streptomycin, at 37 °C in an incubator, in 5% carbon dioxide/95% air and humidified environment. The cell culture medium was changed every two days, and cells were subcultured before reaching confluence. All cell culture reagents were purchased from Gibco (Alfagene, Lisbon, Portugal). Cell handling and manipulation for furthers studies was performed as described in Severino et al. [34].

#### 2.4.2. Alamar Blue Cell Proliferation Assay

Stock solutions of all squaraine dyes were prepared in dimethyl sulfoxide in the millimolar range and stored at 4 °C. These stock solutions, the dyes in the study, were diluted (in different concentrations) in FBS-free culture medium before each experiment. Caco-2 and HepG2 cells were cultured in 96-well culture microplates (at a density of 5 × 10^4^ cells/mL; 100 µL/well) for 24 h, as described in the literature [34,35]. After 24 h for adherence, the culture medium was replaced by solutions of the dyes used in study (0.1, 1.0, 2.5 and 5.0 µM), prepared in FBS-free culture medium. The negative control consisted of exposing the cells only to the FBS-free culture medium. The higher concentration of dimethyl sulfoxide, used in cell viability assays, did not produce statistically significant effects on cell proliferation (data not shown). After incubating the cells with dyes solution for 24 h, cells were exposed to irradiation using three different conditions: (i) 0 min of irradiation (no irradiation), (ii) 7 min of irradiation and (iii) 14 min of irradiation, with light-emitting diode systems that were chosen according to the maximum absorption wavelength of the squaraines in culture media. The irradiation was performed at room temperature, protected from ambient light. After this procedure, cells were returned to the CO_2_ incubator, and incubated for a further 1 h or 24 h with irradiated dyes. Then, the culture media was removed, cells were gently washed with phosphate-buffered saline (pH 7.4), and cell viability was assessed.

The *in vitro* photocytotoxicity of the squaraines was evaluated by quantifying the extent of the reduction of Alamar Blue, by adding 100 µL/well of Alamar Blue solution (10% v/v, diluted in FBS-free culture medium). Absorbance (at 570 and 620 nm) was read, after 5 h of Alamar Blue incubation, using a Multiskan EX microplate reader (MTX Labsystems, FL, USA). Cell viability was calculated by the percentage of Alamar Blue reduction and expressed as a percentage of control (non-exposed cells, 0 µM of squaraine dyes) in each condition, using the equations as reported in the literature [34,35].

Data and statistical analysis: results are expressed as mean value ± standard error of the mean. Statistical significance was determined using a Student’s *t*-test between the data of dye treatments and control, and differences were considered statistically significant when p-value was lower than 0.05. The half-maximal inhibitory concentration (IC_50_) values were calculated by sigmoidal fitting analysis considering a 95% confidence interval and with the bottom of the curves constrained to greater than 0% of relative cell proliferation. All data shown are representative of at least three independent experiments.

#### 2.4.3. Light-Emitting Diode System Specifications

The light-emitting diode exposure device was placed over a 96-well culture cell plate, with the lamps facing the cells, illuminating a single well with a single light emitter from distance of, approximately, 15 mm. All the emitters used in the construction of these irradiation devices were individually characterized in the same conditions and with a supply current of 20.0 mA. These light-emitting diodes have clear, protective epoxy lenses with a diameter of 5 mm and light-emission viewing angle of ± 30°. The emission peaks were measured with an S350 Optometer coupled with an S5124A sensor and an S2575 integrating sphere (UDT Instruments, San Diego, CA, USA). The device was built using aluminum gallium arsenide light-emitting diodes (Roithner, Vienna, Austria) emitting at λ = 652.2 ± 0.7 nm, with a full width at half maximum of Δλ = 21.6 ± 0.3 nm and radiant flux of P = 3.4 ± 0.3 mW.

## 3. Results

### 3.1. Photophysical Parameters

As previously mentioned, an ideal photosensitizer should have photophysical properties such as high absorptivity in the visible and near-infrared regions, the spectral range where penetration of tissues by light is increased, as well as high singlet oxygen production ability, the most critical reactive oxygen species involved in this therapeutic strategy [36,37].

**Figure 2:**
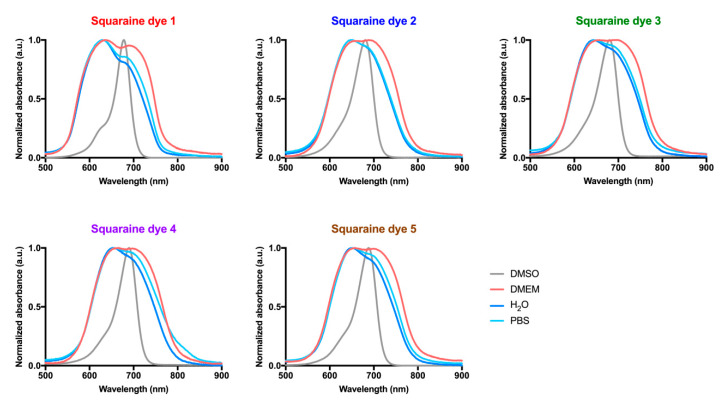
Absorption spectra of dicyanomethylene squaraine dyes **1**–**5** in dimethyl sulfoxide (DMSO), serum-free Dulbecco’s Modified Eagle Medium (DMEM), water, and phosphate-buffered saline (PBS). Absorbance was normalized and is present as arbitrary units (a.u.).

The ground state absorption spectra of the squaraine dyes **1**–**5** were studied in dimethyl sulfoxide (Figure 2), reaching values between 678 and 689 nm (Table 1), which are within the so-called “phototherapeutic window” values. All the synthesized squaraine dyes presented similar and high molar extinction coefficients, and with maximum absorption wavelength in a narrow range, properties reported as preferable for their potential application as photodynamic therapy photosensitizers [38,39].

Absorbance spectra of squaraine dyes in FBS-free DMEM (Figure 2), the aqueous medium used in the biological assays, were also performed to construct light-emitting diode systems suitable for irradiation of the compounds when in contact with the cells. When dissolved in this culture medium, the studied dyes showed broader bands with two distinct maximum absorption peaks, a behavior justified by the formation of aggregates in aqueous media [12,40]. By comparing the spectrum obtained in dimethyl sulfoxide, against those obtained in the culture medium, it can be seen that the novel band formed is absorbing at lower wavelengths than those of the dye’ monomer, indicating the formation of blue-shifted H-aggregates.

**Figure 3:**
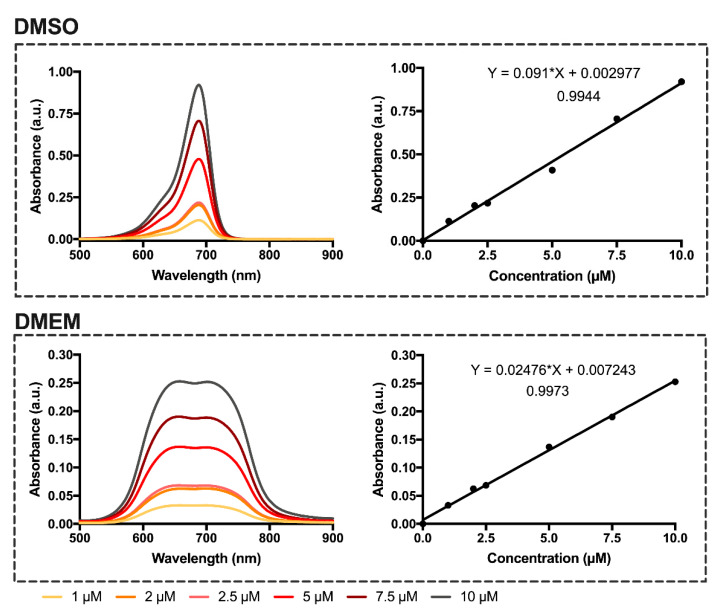
Absorption spectra of dicyanomethylene squaraine dye **5** in dimethyl sulfoxide and serum-free Dulbecco’s Modified Eagle Medium (DMEM) at concentration range 1–10 µM. Linear regression of the dyes’ monomer absorbance as a function of concentration in dimethyl sulfoxide and cell culture medium, equation of the lines and correlation coefficient. Absorbance is present as arbitrary units (a.u.).

In order to verify whether this behavior also occurred in other aqueous media, the spectra of the evaluated dyes were obtained in the water and phosphate-buffered saline (Figure 2). It was observed that in these aqueous media, there was also the formation of H-aggregates and, consequently, dimers of the studied dyes. However, interestingly, the absorbance of the blue-shifted band is usually higher than that of the band corresponding to the monomer, indicating that, in phosphate-buffered saline and water, the equilibrium tends to increase the dye dimerization. In the cell culture medium, the intensity of the bands corresponding to the dimer and monomer are similar, so the equilibrium is achieved when the proportion of free dye and dimer is similar. Despite these spectral changes, the dyes displayed great absorption (4.19 < log ε < 4.49) within the tissue transparency spectral region in all studied aqueous media (Figure 3 and Table 1).

**Figure 4:**
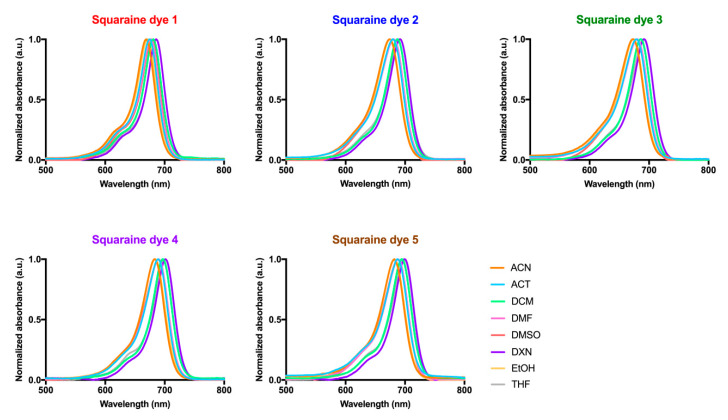
Absorption spectra of dicyanomethylene squaraine dyes (**1**–**5**) in acetonitrile (ACN), acetone (ACT), dichloromethane (DCM), dimethylformamide (DMF), dimethyl sulfoxide (DMSO), 1,4-dioxane (DXN), ethanol (EtOH) and tetrahydrofuran (THF). The absorbance was normalized and is presented as arbitrary units (a.u.).

The absorption behavior of dyes in several organic solvents of different polarities was also evaluated (Figure 4 and Table 1). Regardless of the solvent in which the dyes were solubilized, these compounds displayed narrow bands in the red and near-infrared regions (maximum absorption wavelength values between 668–700 nm) and evidenced high molar extinction coefficient values (4.91 < log ε < 5.27) in a settlement with π–π* transitions. Although there is no linearity in the maximum absorption wavelength values of the dyes with the polarity of the solvents, it should be noted that in ethanol, one of the most polar organic solvents used, all dyes showed lower maximum absorption wavelengths, and these were higher in 1,4–dioxane. This photophysical property was previously studied for these dyes in methanol and in chloroform, more and less polar solvents, respectively, in which absorption wavelengths were found within the expected and verified in the organic solvents studied in the present work. In accordance with those as mentioned above, a blue shift is again noticeable the higher the polarity of the solvent used, since all dyes showed lower maximum absorption wavelengths in methanol than in ethanol. Contrary to what is observed in aqueous media, in all organic solvents, the formation of a hypsochromic shoulder is found, a photophysical property inherent to this dyes’ family.

Establishing a relationship between the structure of the dyes and their absorption ability, it is possible to verify that the dye possessing two heterocyclic indolenine moieties (**1**) was the one that evidenced being absorbed at lower wavelengths in most solvents, compared to the other dyes. Among benzothiazole-derivatives (**2**–**4**), the iodination of one of the heterocycle units (**4**) favored this photophysical property, since it allowed a red shift of the maximum absorption wavelength of about 10 nm in all organic solvents.

The determination of singlet oxygen quantum yields of the synthesized dyes is of high relevance in the approach of this work since this reactive oxygen species is known for its high cytotoxicity and for causing significant cell damage, triggering cell death [41]. Moreover, the evaluation of this photophysical parameter allows us to deduce the types of reaction produced by the photosensitizing molecules [11], since the triplet state of a photosensitizer, as generally stated in the introduction, can produce reactive oxygen species by two divergent mechanisms: type I reaction mechanism, in which the photosensitizer in the triplet state react with organic substrates to produce free radicals that interact with oxygen to generate, for example, superoxide radical anions, and a type II reaction mechanism, in which the triplet state energy is directly transferred to ground-state triplet oxygen to produce singlet oxygen [3,42]. This determination was performed in chloroform, and phenazine (singlet oxygen quantum yield of 0.84 [33,43]) was used as standard (Table 1). Due to the extremely reduced intensity of the phosphorescence emissions of singlet oxygen in common solvents, such as ethanol or acetonitrile, we were only able to record this emission in chloroform, despite the high sensitivity of the detector used.

### 3.2. Photostability Monitoring

Photostability is defined as the response of a drug or a pharmaceutical product to light exposure (e.g., sunlight, ultraviolet or visible radiation) [44]. Many of the medicines commercially available on the market are sensitive to radiation and, therefore, can be degraded during the process of their manufacture, storage, or administration. In this way, photostability studies are of great importance, since degradation of the potential drug may result in loss of its potency, efficacy, or the production of side effects when administered [44,45,46]. The relevance of the photostability determination of photosensitizer candidate compounds is praised for the fact that it is desired to ensure that the cytotoxic effect is produced exclusively by the photosensitizing molecule without interference from degradation products [47].

**Figure 5:**
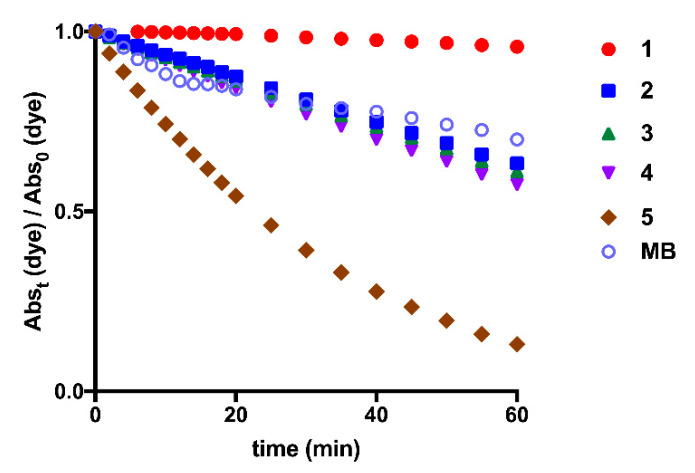
Photostability evaluation of dicyanomethylene squaraine dyes **1**–**5** and methylene blue (**MB**) in dimethyl sulfoxide (Abs_t_ (dye)—absorbance of squaraine dye when irradiated at time t; Abs_0_ (dye)—absorbance of squaraine dye without light-emitting diode irradiation).

For the photostability monitoring, solutions of each squaraine dye in dimethyl sulfoxide were irradiated with a suitable light-emitting diode system, which was chosen according to the maximum absorption wavelength of the squaraines in this solvent (Figure 5). Dicianomethylene squaraine dyes **1**–**5** showed great light-stability, to emphasize dye **1**. Benzothiazole-based squaraine dyes, regardless of the *N*–alkyl groups attached to it, showed similar photostability levels. Interestingly, an antagonistic effect on the stability produced by the benzoselenazole-derived dye **5** is verified. Compared to methylene blue, a commercial photosensitizing molecule widely used in clinical trials [48,49], benzothiazole derivatives **2**–**4** showed similar stability, the indolenine-based squaraine dye **1** evidenced improved light-stability and the dye bearing benzoselenazole moiety **5** presented poor stability to this physical agent. As a consequence of the lower photostability of this last compound, photofading was also visible, since the ability to emit coloration was lost during the period of radiation exposure.

### 3.3. Cell Viability Photodynamic Effects

Ideally, a photosensitizer compound should exhibit characteristics such as low dark-cytotoxicity and high cytotoxicity when exposed to light with a suitable wavelength. Thus, to evaluate the photocytotoxicity of the squaraine dyes **1**–**5**, HepG2 and Caco-2 cells were used as *in vitro* models. These cell lines have ideal characteristics for their application in cytotoxicity assays, mainly because they are adherent epithelial cells, avoiding the loss of cells during the washes and consequent errors in the assessment of cell viability, as well as allowing the analysis of hepatic toxicity and their potential use as photodynamic therapy photosensitizers in the treatment of colorectal cancer. Thus, cells were incubated with a set of solutions with different dye concentrations for 24 h, then were irradiated with an adequate light-emitting diode system (for 7 or 14 min), and after 1 or 24 h of contact with irradiated dyes, the cells’ viability was assessed by the Alamar Blue method [35,50].

**Figure 6:**
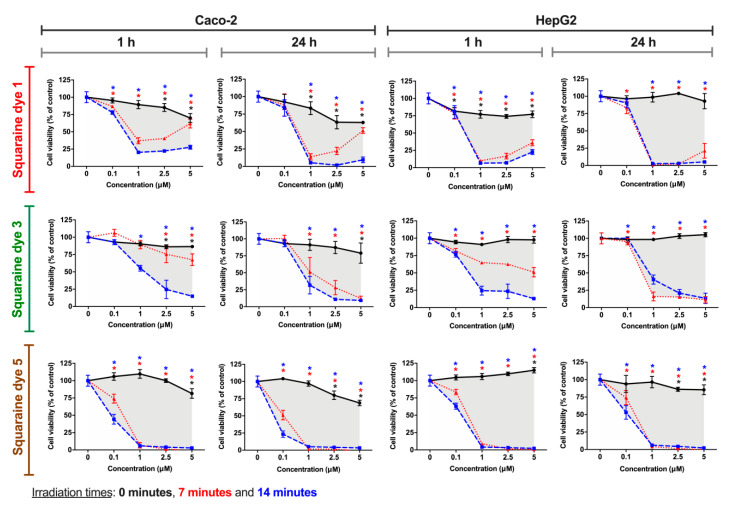
Photodynamic effects of dicyanomethylene squaraine dyes **1**, **3** and **5** on Caco-2 (human colorectal adenocarcinoma) and HepG2 (human hepatocellular carcinoma) cells. After cells’ incubation for 24 h with the respective dye (at 0.1, 1, 2.5 and 5 µM), cells were irradiated with suitable light-emitting diode systems for 0, 7, and 14 min (black, red and blue lines, respectively). After irradiation, the cells were maintained in contact with the irradiated dyes for 1 h or 24 h, and Alamar Blue reduction assay was performed for cell viability assessment. Results are expressed as % of control, non-treated cells, and presented as mean ± standard error of the mean (n = 3 independent experiments; each experiment in quadruplicates). Conditions with statistical significance compared to the negative control (*p*-value < 0.05) are indicated by an asterisk (*) of the color relative to the irradiation time. The shaded area delimits the difference in the percentage of cell viability between treatment with and without irradiation.

Although the cytotoxic effects are visibly dependent on the cell line under study, all squaraine dyes, generally, have been shown to exhibit more significant cytotoxicity after irradiation than in the dark (non-irradiated), regardless of the incubation time with the irradiated dye. Considering the dicyanomethylene squaraine dyes that showed a more significant phototherapeutic effect, Figure 6 presents the cell viability results for cells exposed to compounds **1**, **3** and **5**.

Dye **1** demonstrated photodynamic activity in both tumor cell lines, at 1 and 2.5 µM, in Caco-2 cells, and at tested concentrations equal to or greater than 1 µM, in HepG2 cells. Interestingly, in Caco-2 cells, there is a slight cell viability increase at 2.5 and 5 µM of the dye when irradiated for 7 min, a behavior that can be explained by the generation of a protective cellular mechanism when exposed to certain doses of free radicals and other reactive oxygen species [51,52,53]. The *in vitro* phototherapeutic effects produced by dye **3** are most evident at 1 µM or higher concentrations when irradiated for 14 min at both exposure times. Finally, the dye that proved to be more potent and at the same time with better photodynamic activity was dye **5**, since it caused a decrease in cell viability >30% (threshold level for cytotoxicity according to the Generally Recognized As Safe standards [54]) at all the tested concentrations only under irradiated conditions. The potency of this compound, among the dicyanomethylene derivatives, is supported by this being the one with the highest singlet oxygen quantum yield (0.08), as well as having moderate photostability.

**Figure 7:**
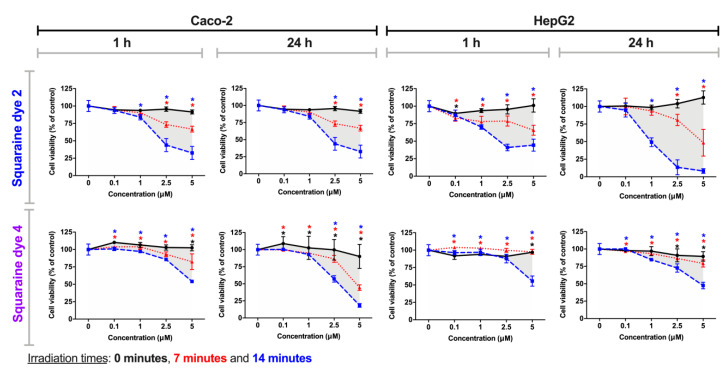
Photodynamic effects of dicyanomethylene squaraine dyes **2** and **4** on Caco-2 and HepG2 cells. After cells incubation for 24 h with the respective dye (at 0.1, 1, 2.5 and 5 µM), cells were irradiated with suitable light-emitting diode systems for 0, 7, and 14 min (black, red and blue lines, respectively). After irradiation, the cells were maintained in contact with the irradiated dyes for 1 h or 24 h, and Alamar Blue reduction assay was performed for cell viability assessment. Results are expressed as % of control, non-treated cells, and presented as mean ± standard error of the mean (n = 3 independent experiments; each experiment in quadruplicates). Conditions with statistical significance compared to the negative control (*p*-value < 0.05) are indicated by an asterisk (*) of the color relative to the irradiation time. The shaded area delimits the difference in the percentage of cell viability between treatment with and without irradiation.

Dyes **2** and **4**, in contrast to the other studied compounds, did not produce significant photocytotoxicity at the lowest concentrations (0.1 and 1 µM), with reductions in cell viability exceeding 30% at concentrations of 2.5 and 5 µM (except for treatment in the HepG2 cell line with dye **2**, after 24 h of contact with irradiated dyes) (Figure 7). The effect of the irradiation time of these dyes is to be highlighted, since after 14 min of irradiation, cell viability is considerably lower than only after 7 min of irradiation. Even so, this observation is verified in a more marked way also at the highest concentrations. The incubation time with irradiated dyes, with the exception of dye **2** for the HepG2 cell line, does not seem to influence the variation in cell viability. Thus, in general, these benzothiazole-based dyes showed a lower antiproliferative potency under the tested conditions.

The determined IC_50_ values, an important measure of potency for a given agent that represents the concentration at which a substance exerts half of its inhibitory effect, are presented in Table 2. Interestingly, for dicyanomethylene squaraine dyes **1**, **3** and **5**, the IC_50_ values are >5 µM in both tumor cell lines, in the absence of light but significantly lower when irradiated. Among these last three dyes, squaraine dye **5** stands out since it shows IC_50_ values in irradiated conditions always less than 0.2 µM, lower than the values obtained among the studied dyes, and which show the high photodynamic potency of this photosensitizer candidate. In agreement with those reported above, dicyanomethylene squaraines dyes **2** and **4** showed low cytotoxicity at both exposure times to irradiated dye, except when irradiated for 14 min, a condition in which there is a moderate decrease in the IC_50_ values.

## 4. Discussion

One of the most significant barriers to be overcome concerning photodynamic therapy is the number of clinically approved photosensitizers since only in recent years has there been an increased demand for novel molecules with ideal properties for their use in this therapeutic strategy. Besides, commercially available photosensitizers have flaws, including the fact that they cause high skin photosensitivity, which chemistry, cellular and molecular biology, as well as oncology research areas, have been joining their efforts in order to overcome. An example of this are the studies reported in recent years in the sense of finding ideal squaraine cyanine dyes for their application in this therapeutic strategy, which involve the evaluation of their photophysical, photochemical and photobiological properties, and which have highlighted that these dyes’ core, with the suitable substituents, may have high potential about its application as a photodynamic therapy anti-cancer agent [31,40].

All studied dyes showed low singlet oxygen quantum yields. Despite this, it is observed that, among the evaluated dyes, the non-symmetrical dye bearing a benzoselenazole heterocycle moiety **5** was the one that showed the highest quantum yield (0.08). Among the benzothiazole-based compounds, what stood out the most concerning to this photophysical property was the dye iodinated at position 6 of one of the heterocycle units **4** (0.06). The better performance of these dyes is mainly due, as already mentioned in the literature [11,32], to the presence of heavy atoms in their structure, a structural modification that improves their ability to produce this reactive oxygen species. The esterification of the benzothiazole-based’ *N*–alkyl chain dye did not prove to be beneficial concerning this property. 

The moderate to excellent photostability of dicyanomethylene squaraine dyes is possibly due to the fact that these synthesized compounds are derived from indolenine, a heterocyclic ring which, according to Terpetschnig et al. [55], enhances their stability and biological application to this core of dyes. In agreement with that, it was verified that the most light-stable dye involved in this study was the one possessing two units of indolenine heterocycle, a compound that proved to be less efficient in the production of singlet oxygen. The introduction of benzothiazole and benzoselenazole heterocycles led to the increased sensitivity of these compounds to the luminous agent. Thus, this study also confirms the relevance of the chosen heterocycle ring in this photochemical property, since dyes with the same heterocyclic-structure show similar light-stability. Also, until 14 minutes of irradiation, we found that none of the dyes has a degradation level greater than 30%, so, even if the degradation products may induce antiproliferative effects, the levels of cytotoxicity will mostly come from the dyes’ activity.

The results of phototherapeutic treatments in the Caco-2 and HepG2 cell lines confirm the high photodynamic efficiency of dicyanomethylene-based squaraine dyes. Dyes **1**, **3** and **5** were those that stood out the most, while the rest had low photocytotoxicity. Among the three best dyes, dye **5** stands out, from which it can be concluded that the introduction of the benzoselenazole heterocycle induced an increase in the potency of this dyes’ core since it was the only one to induce considerable photodynamic activity at the lowest concentration tested on the Caco-2 cell line. From the dyes derived from benzothiazole, the esterification of the *N*–alkyl chain and the iodination of this heterocycle resulted in the decrease of their phototherapeutic efficiency, since they only presented cytotoxicity at the highest concentrations tested.

As cited in the introduction, Pandey et al. [29] mention that candidate compounds for photosensitizers that have good affinity and interaction with serum albumin are more likely to exhibit desired photosensitizing properties. In the present study, it was proved that, for this core of dyes in the conditions and cell lines tested, this fact was not so linear. Although the symmetrical dye **1** in our fluorescence study presented a high affinity for human and bovine serum albumin and in this photodynamic study it showed high photocytotoxicity, for dyes **3** and **5**, despite showing good phototherapeutic effect, their interaction with these biomolecules were weak to moderate. Also, in disagreement, it was the fact that the good affinity of dye **3** with serum proteins did not result in improved photodynamic activity.

This study, which is in line with some of our previous studies, in addition to deciphering the impact of specific structural changes on the physicochemical and biological properties of this core of dyes, aims to increase the range of molecules that photosensitizer candidates of colorectal and hepatocellular cancers, the third leading cause of cancer death worldwide and the most prominent liver malignancy [56,57], respectively, which are, therefore, health problems of significant impact in today’s society. The dicyanomethylene squaraine dyes with substantial *in vitro* phototherapeutic activity presented in this work (**1**, **3** and **5**), in comparison with the photodynamic studies in aminosquaraine dyes that we had previously reported also in the Caco-2 and HepG2 cell lines [21,40], in summary, performed less dark cytotoxicity, as well as having equal to greater antiproliferative effects after being irradiated. The improved effectiveness of the compounds herein reported is evidenced by the IC_50_ values, in which the aminosquaraine dyes scarcely exhibit values below 1 µM [40], while the dicyanomethylene derivatives produce enough cytotoxicity to reach IC_50_ values below 0.5 µM.

The general analysis of the results obtained in this research allows us to affirm that dyes derived from dicyanomethylene exhibit characteristics favorable to their potential application as cancer photodynamic therapy photosensitizers, as well as to point out that the main mechanism of cytotoxicity production of these compounds are type I reactions since the singlet oxygen quantum yields indicate the low output of this reactive oxygen species.

## 5. Conclusions

In conclusion, we have assessed the singlet oxygen production ability, photostability, and the *in vitro* photoactivation dicyanomethylene squaraine cyanine dyes in the Caco-2 and HepG2 tumor cell lines. All dyes displayed high absorption within the so-called “phototherapeutic window”. The dyes under study showed singlet oxygen quantum yields within the range of 0.01 and 0.08, levels considered low. It has further been found that the bleaching of the dicyanomethylene-derived dyes is related to the number of units of the indolenine heterocycle ring since the dye with two units of this heterocycle was the one that exhibited the best stability results. *In vitro* photocytotoxicity studies showed that dicyanomethylene-based squaraine dyes are potential candidates for cancer photodynamic therapy photosensitizers since none of the dyes showed excessive cytotoxicity in the absence of radiation. It can also be concluded that the photodynamic effect produced by the most promising dyes should be mostly due to type I reactions, given the relatively low singlet oxygen quantum yields. The results of this work showed that most of the studied squaraine dyes, in particular **5**, despite being relatively photosensitive, presented a promising photodynamic activity.

## Figures and Tables

**Table 1:** Summary of data obtained in the visible and near-infrared spectroscopy of dicyanomethylene squaraine dyes **1**–**5** in acetonitrile (ACN), acetone (ACT), dichloromethane (DCM), dimethylformamide (DMF), dimethyl sulfoxide (DMSO), 1,4-dioxane (DXN), ethanol (EtOH), tetrahydrofuran (THF), serum-free Dulbecco’s Modified Eagle Medium (DMEM), water and phosphate-buffered saline (PBS) (λ_max_ in nm). Singlet oxygen quantum yields (Φ_Δ_) of the dyes in chloroform (CFM), determined using phenazine as standard.

Solvent	Dye 1		Dye 2		Dye 3		Dye 4		Dye 5	
	λ_max_	log ε	λ_max_	log ε	λ_max_	log ε	λ_max_	log ε	λ_max_	log ε
Organic Solvent										
ACN	668	5.05	673	4.93	672	5.04	683	5.07	681	5.06
ACT	674	5.05	680	4.94	678	4.98	688	5.06	687	5.03
DCM	680	5.20	686	5.03	685	4.96	696	5.11	694	5.20
DMF	676	5.04	681	4.92	679	4.96	689	5.04	688	5.03
DMSO	681	5.27	680	5.11	678	5.16	688	5.20	689	4.96
DXN	685	5.03	691	4.97	692	5.01	700	5.06	699	5.08
EtOH	671	5.05	674	4.92	673	4.98	683	5.02	682	5.03
THF	685	5.05	691	4.91	691	4.97	700	5.07	698	5.06
Aqueous Solvent										
DMEM ^1^	693	4.38	697	4.37	703	4.39	710	4.19	707	4.39
H_2_O ^1^	682	4.46	688	4.43	689	4.46	693	4.29	696	4.49
PBS ^1^	683	4.40	688	4.42	686	4.44	695	4.23	689	4.47
Singlet Oxygen Quantum Yield (Φ_Δ_)										
CFM	0.01		0.03		0.03		0.06		0.08	

^1^ The maximum absorption wavelength values shown correspond to those of the respective dye’ monomer.

**Table 2:** *In vitro* cytotoxicity activity (IC_50_ values, µM) of dicyanomethylene squaraine dyes **1**–**5** against Caco-2 and HepG2 cells exposed to different irradiation times (0, 7 and 14 min) and different incubation periods with the irradiated dyes (1 and 24 h).

Dye	Irradiation Time (min)	Caco-2		HepG2	
		1 h	24 h	1 h	24 h
**1**	0	>5	>5	>5	>5
	7	0.173	0.536	0.285	0.241
	14	0.563	0.244	0.259	0.263
**2**	0	>5	>5	>5	>5
	7	>5	>5	>5	>5
	14	2.520	1.426	1.432	1.016
**3**	0	>5	>5	>5	>5
	7	>5	0.983	3.661	0.768
	14	1.306	0.345	0.116	0.155
**4**	0	>5	>5	>5	>5
	7	>5	>5	>5	>5
	14	>5	2.943	>5	4.935
**5**	0	>5	>5	>5	>5
	7	0.193	0.094	0.263	0.188
	14	0.079	0.032	0.143	0.108
